# Comment on “Laser-induced modulation of root dentin surface for growth factor release and enhanced stem cell response”

**DOI:** 10.1007/s10103-026-05068-z

**Published:** 2026-10-15

**Authors:** Rodrigo Farias

**Affiliations:** 1 NEOSC, São José, Brazil; 2 Universidade Federal de Santa Catarina, Florianópolis, Brasil

**Keywords:** Laser irradiation, Dentin, TGF-β1, Regenerative endodontics, Stem cells, Laser dosimetry, Tissue regeneration

## Abstract

Background: Laser conditioning of dentin has been investigated as a strategy to enhance growth factor release and cellular responses in regenerative endodontics. The recent study by Mokhtari et al. reported laser-induced modulation of root dentin, increased transforming growth factor-beta 1 (TGF-β1) release, and enhanced alkaline phosphatase (ALP) activity. However, several methodological aspects should be considered when interpreting these findings and their potential clinical translation. Main text: A critical analysis identifies important limitations in the experimental design. First, all laser-treated specimens were previously exposed to sodium hypochlorite, without a laser-only control, preventing the independent determination of the effect attributable to laser irradiation. Second, the concomitant increase in TGF-β1 release and ALP activity demonstrates an association but does not establish a causal mechanistic relationship, as no inhibition or neutralization experiments targeting TGF-β1 signaling were performed. Third, the use of mature dentin obtained from adult teeth limits direct extrapolation to immature permanent teeth, which are the clinical context emphasized in regenerative endodontics. In addition, the use of stem cells from human exfoliated deciduous teeth (SHED) does not fully represent endogenous cell populations involved in regenerative endodontics, and cellular migration or chemotaxis was not evaluated. Finally, differences in power and exposure time among wavelengths, together with the absence of calibrated power measurements and direct temperature monitoring, limit interpretation of dosimetry, dose-response relationships, and thermal safety. Conclusion: The findings provide preliminary evidence that laser irradiation may modify dentin and influence growth factor release and cellular responses. Nevertheless, laser-only controls, mechanistic assessment of TGF-β1 signaling, immature dentin models, evaluation of cellular migration, calibrated laser dosimetry, and temperature monitoring are necessary to strengthen the mechanistic and translational evidence supporting laser-assisted regenerative endodontic applications.

Dear Editor,

We read with interest the article by Mokhtari et al. entitled “Laser-induced modulation of root dentin surface for growth factor release and enhanced stem cell response,” recently published in Lasers in Medical Science [1]. The study provides relevant experimental data concerning laser conditioning of dentin, TGF-β1 release, and stem-cell responses.

However, several methodological considerations may affect the interpretation and translational extrapolation of its findings.

First, all laser-treated specimens were previously exposed to 1.5% sodium hypochlorite (NaOCl), whereas no laser-only group was included [1]. Consequently, the specific contribution of laser irradiation cannot be independently determined. The observed changes in TGF-β1 release and ALP activity may reflect the combined effects of NaOCl and laser treatment. Therefore, comparisons with the NaOCl/EDTA group cannot establish that laser irradiation alone produces an effect equivalent or superior to EDTA. A laser-only control would be necessary to isolate the effect attributable specifically to irradiation.

Second, the concomitant increase in TGF-β1 release and ALP activity should be interpreted as an association rather than a causal relationship. No inhibition or neutralization experiment was performed to determine whether the cellular response depended on TGF-β1 signaling. Thus, the findings support an association between TGF-β1 release and early cellular differentiation, but they do not establish a direct mechanistic pathway.

Third, the translational relevance of the dentin model deserves consideration. The study is framed within regenerative endodontic procedures for immature permanent teeth, whereas the experimental specimens were obtained from mature single-rooted teeth from adult patients [1]. Because TGF-β1 distribution and content may differ according to the developmental stage of the tooth, extrapolation from mature dentin to immature permanent teeth should be made cautiously [2].

The use of SHED also represents an important limitation in terms of cellular-model representativeness. SHED provide a useful in vitro mesenchymal stem-cell model, but they may not reproduce the behavior of endogenous cell populations relevant to regenerative endodontics, including SCAP and DPSC. Moreover, the study did not assess cell migration or chemotaxis. Previous work has demonstrated that dentin conditioning can influence dental pulp stem-cell migration and differentiation [3]. Therefore, increased TGF-β1 release should not be interpreted as evidence of enhanced recruitment of endogenous regenerative cells.

Finally, laser dosimetry warrants further consideration. The reported energy densities were achieved using different power and exposure-time combinations among wavelengths, potentially introducing a time-dependent thermal component. In addition, the absence of calibrated power measurements and direct temperature monitoring limits the interpretation of dose-response relationships and thermal safety, particularly in the context of immature teeth with thinner dentinal walls.

Overall, the study contributes useful preliminary evidence regarding laser-induced dentin modification. Nevertheless, laser-only controls, mechanistic evaluation of TGF-β1 signaling, immature dentin models, direct assessment of cellular migration, calibrated dosimetry, and temperature monitoring would strengthen the evidence required for clinical translation.

Yours sincerely,

[Authors]

## Data Availability

No datasets were generated or analysed during the current study.
